# Television-based health promotion in general practice waiting rooms in London: a cross-sectional study evaluating patients’ knowledge and intentions to access dental services

**DOI:** 10.1186/s12903-016-0252-6

**Published:** 2016-07-20

**Authors:** Mohammed Jawad, Sam Ingram, Imran Choudhury, Anne Airebamen, Kostakis Christodoulou, Amanda Wilson Sharma

**Affiliations:** Department of Primary Care and Public Health, Imperial College London, London, W6 8RP UK; Independent researcher, London, UK; Public Health Consultant, NHS Brent, London, UK; Health Improvement Specialist, NHS Brent, London, UK

**Keywords:** General practice, Health promotion, Television, Audio-visual, Family health

## Abstract

**Background:**

This study aimed to evaluate whether television-based dental health promotion initiatives in General Practice waiting rooms would increase patients’ knowledge of and intentions to seek dental services.

**Methods:**

This cross-sectional survey of 2,345 patients attending 49 General Practices in Brent, northwest London, evaluated the ‘Life Channel’ – a series of six brief health promotion advertisements, including one dental health promotion advertisement, displayed over ten minutes on television in General Practice waiting rooms. Primary outcome measures were a self-reported gain in the knowledge to contact a National Health Service (NHS) and emergency dentist, and an intention to seek dental services, attributed to viewing the Life Channel.

**Results:**

Among the 1,088 patients who did not know how to contact an NHS dentist prior to the survey, and the 1,247 patients who did not know how to contact an emergency dentist prior to the survey, 48.0 % (95 % CI 45.0-51.0 %) and 35.1 % (95 % CI 32.4-37.8 %) attributed the Life Channel to educating them how to do so, respectively. Among the 1,605 patients who did not have any intention to contact a dentist prior to the survey, 15.2 % (95 % CI 13.4-17.0 %) attributed the Life Channel to creating such an intention. We report adjusted odds ratios on sociodemographic disparities in this evaluation.

**Conclusions:**

Television-based dental health promotion may significantly increase knowledge of and intention to seek dental services in this sample in London. Television-based dental health promotion may appeal more to certain population groups. More research is needed to identify longer term outcomes of television-based health promotion.

## Background

General Practice waiting rooms are being increasingly seen as a health promotion medium. Considering that health promotion discussions within consultation rooms may be time-consuming for physicians [1] and that health promotion in waiting rooms can be associated with increasing levels of patient satisfaction [2, 3], these are promising locations in which to conduct health promotion activities.

There is conflicting evidence as to the effectiveness of health information posters in the waiting room of General Practices. In one Manchester-based General Practice 82 % of 319 patients reported observing posters and remembering the subject on the display [4]. Despite over 50 % expressing a want for further information, no data were gathered as to whether such recall had impacted on health behaviour. A similar study found that 22 % of patients observed information on noticeboards, and that recall of health information decreased as the number of health topics increased [5]. One study from the US concluded that educational posters to reduce antibiotic use did not result in a significant change in antibiotic prescriptions [6].

Novel health promotion approaches in the clinical waiting room go beyond traditional methods such as noticeboards and posters, and include screening for common behavioural risk factors [7], directly encouraging discussion of specific health topics with a healthcare professional [8], and self-monitoring blood pressure [9]. Audio-visual health promotion are also used, and are suitable for those with low literacy skills or impairments over an ability to read [10]. Several studies from a variety of Western healthcare settings report that using audio-visual health promotion messages can better teach patients about their condition, increase confidence in self-management, and promote active communication and shared decision making with physicians [3, 11]. Specifically to General Practice settings, studies from French [12] and Belgian [13] general practitioners have suggested that clinical waiting rooms are an acceptable and useful medium for health promotion; the former study placing particular importance on the effects of television health promotion [12]. Another Belgian study showed that broadcasting audio-visual health information about vaccinations was significantly associated with increased adult tetanus booster prescriptions [14]. However one randomised controlled trial in a US diabetic waiting room clinic found no difference in diabetes self-management between patients receiving educational brochures and patients receiving a computer multimedia education programme [15]. Another randomised controlled in a rheumatology waiting room clinic found no difference in patient multidisciplinary team attendance between patients exposed to television-based health promotion and patients receiving no health promotion [16].

Dental health promotion has received little attention outside dental practices, and there is a paucity or research regarding the effectiveness of dental health promotion strategies in General Practice waiting rooms. This is of concern in light of the 2009 Adult Dental Health Survey which reported that only 58 % of adults in the United Kingdom (UK) said they had tried to make an NHS dental appointment in the last 3 years and only 17 % of dentate adults had very healthy periodontal (gum) tissues and no periodontal disease [17]. As of January 2013, approximately 50 local governments in the UK adopted the ‘Life Channel’, a fee-commissioned service which displays a series of television-based health promotion advertisements (www.thelifechannel.co.uk). In our local area, one of these advertisements focused on the importance of dental health. Our aim was to evaluate this service as it is unknown the extent to which such television-based health promotion initiatives are effective at changing knowledge and intentions to seek dental services.

## Methods

### Design, setting and sampling

We conducted a cross-sectional survey of patients in General Practices in the London Borough of Brent, the most ethnically diverse and one of the most deprived boroughs in the United Kingdom [18]. The sample frame was all General Practices that had a television in the waiting area and showed the Life Channel. The Life Channel consists of six health promotion television advertisements (dental health, smoking cessation, chlamydia, contraception, HIV/hepatitis and influenza), a full cycle of which is shown over a ten minute period interspersed with commercial advertising. The dental health advert, which lasted approximately 80 s and was targeted to adults, stated that everyone should visit a dentist at least every 2 years, and gave details of how to contact both NHS and emergency dentists. Of all 68 General Practices in Brent, 49 (75.1 %) showed the Life Channel. We contacted these 49 General Practices, inviting them to participate in an anonymous, interviewer-administered survey. All General Practices agreed to participate.

The study took place between February and March 2013 (five weeks). Eighteen trained facilitators visited each General Practice at least once, the time of which occurred according to facilitator suitability and not at random. After taking informed consent they sampled all patients in the waiting room; for newly-entered patients they waited at least five minutes before approach, which enabled some viewing of the Life Channel. No staff were sampled and due to the exploratory nature of this study no sample size was calculated (although we conducted a post-hoc power analysis). Patients were able to complete either a paper copy or digital copy of the survey (the latter via a Smartphone). Each survey took approximately five minutes to complete.

### Questionnaire and measures

The survey was a self-developed, pre-piloted but non-validated tool which was originally piloted in Brent to 754 patients in 16 General Practices in 2010. The low completion rate then led to a reduction in the number of questions and a refinement in question wording. The current survey used in this study contained 28 items which covered two main categories. Sixteen questions asked about the Life Channel, including its noticeability, acceptability, and whether it had changed knowledge and intentions about access dental and smoking cessation services. It did not ask any questions about change in knowledge and intentions about the other four health topics seen on the Life Channel (chlamydia, contraception, HIV/hepatitis, and influenza). Twelve questions gathered socio-demographic and health data. The full survey is available upon request.

This study had three main binary outcome measures. The first outcome measure was those who selected “I know how to find an NHS dentist in Brent because of seeing the Life Channel today” after being asked “Which one of the following statements about finding an NHS dentist is most accurate for you?” (other options: “I don’t know how to find an NHS dentist in Brent”, “I know how to find an NHS dentist in Brent because of something else”, and “None of the above”). The second outcome measure was those who selected “I know how to contact a dentist in an emergency from seeing the Life Channel today” after being asked “Which one of the following statements about finding an emergency is most accurate for you?” (other options: “I don’t know how to contact a dentist in an emergency”, “I know how to contact a dentist in an emergency from another source”, and “None of the above”). The final outcome measure was those who selected “I’m planning to contact a dentist because of seeing the Life Channel today” after being asked “Which one of the following statements is most accurate for you?” (other options: “I’m not planning to see a dentist at the moment”, “I’m planning to contact a dentist for another reason”, and “None of the above”). According to the Theory of Planned Behaviour, intention to commit to a health behaviour is regarded as one of the most powerful indicators of behaviour change [19].

Predictor variables included age (29 or under/30 to 39/40 to 49/50 to 59/60 or over), gender, ethnicity (White/South Asian/Black/Mixed or other), education (primary/secondary/college/university/other), the presence of a chronic disease (ascertained by the question “Are your day-to-day activities limited because of a health problem or disability which has lasted, or is expected to last, at least 12 months (including problems related to old age)?), smoking status (non-smoker/ex-smoker/smoker) and whether a dentist had been visited in the last 2 years (yes/no).

### Statistical analysis

Observations with missing data were not removed prior to analysis. The denominator for the first two outcome measures excluded those who knew how to contact an NHS/emergency dentist from another source as this study was only interested in evaluating the service for those who did not already know how to contact an NHS or emergency dentist. Similarly, the denominator for the third outcome measure excluded those who were already intending to contact a dentist for another reason.

We conducted a simple bivariate analysis by cross-tabulating outcome measures by predictor variables. We conducted three forced multi-level logistic regression models to examine which predictors were independently associated with our three outcome measures. These were adjusted for all covariates in addition to the waiting times of each patient, and whether patients could hear the Life Channel. A two-level random-intercept model was used to account for clustering effects around General Practices (Stata command: xtmelogit). We reported adjusted odds ratios (AORs) with a 95 % confidence interval (CI) and took an alpha probability value of 0.05 as statistically significant. We measured the observed power of each model in a post-hoc analysis. Finally, we ran descriptive statistics on other evaluative aspects of the Life Channel (e.g., noticeability, acceptability). All statistical analyses were performed using Stata 12 (StataCorp).

## Results

### Participants

Of 3,055 approached patients, 2,345 (76.8 %) completed the survey. No data on response bias were available. The median (interquartile range) number of responses per practice was 26 (11–40). At the time of the survey, over half of respondents (54.2 %) had been waiting for less than ten minutes, 34.8 % had been waiting for 10–30 min and 11.1 % had been waiting for over 30 min for an appointment with their General Practitioner.

Table 1 presents the characteristics of our sample. Nearly half (48.1 %) of the sample were under the age of 40 years, most (58.8 %) were female and just over a third (34.2 %) were of South Asian ethnicity. The majority had either college (37.9 %) or university (36.9 %) level education. Just over one quarter (28.1 %) reported the presence of a chronic disease, 15.7 % reported themselves as smokers, and 75.3 % reported visiting a dentist in the last 2 years. Comparing our sample demographics to locally available census data (Age: 27 % aged 18–29, 23 % aged 30 to 39, 18 % aged 40 to 49, 14 % aged 50 to 59, 19 % aged 60 or over; Ethnicity: 36 % White, 33 % South Asian, 19 % Black, 12 % Mixed/other ethnicity; Education: 19 % primary, 10 % secondary, 20 % college, 33 % university, 15 % other) [18], we believe we had a representative sample of Brent despite our convenience sampling method. For all logistic regression models described below, a post-hoc power analysis suggested we achieved 99 % power given our sample size, number of predictors and R^2^ value for each model.

**Table 1:** Sample characteristics, and prevalence and predictors of intention to contact a dentist and intention to contact another health service by sociodemographic and health variables

Characteristic		Total sample		Knows how to contact NHS dentist ^a^				Knows how to contact emergency dentist ^b^				Intends to contact a dentist ^c^			
		N	%	N	%	AOR	95 % CI	n	%	AOR	95 % CI	n	%	AOR	95 % CI
Total				522	48.0			438	35.1			244	15.2		
Age (years)	29 or under	454	21.4	111	50.0	1.00	Ref.	76	28.8	1.00	Ref.	54	15.9	1.00	Ref.
	30 to 39	566	26.7	130	45.8	0.78	0.48, 1.27	117	36.0	1.84	1.14, 2.97*	72	18.6	1.32	0.79, 2.21
	40 to 49	471	22.2	103	47.7	0.75	0.44, 1.29	105	42.9	2.11	1.27, 3.50**	44	13.2	0.59	0.32, 1.06
	50 to 59	311	14.7	80	52.3	1.02	0.57, 1.82	67	43.5	2.06	1.13, 3.74*	42	19.3	0.97	0.53, 1.79
	60 or over	321	15.1	63	47.7	1.06	0.56, 2.00	41	23.7	1.35	0.71, 2.55	24	11.1	0.64	0.30, 1.36
Gender	Female	1243	58.8	267	46.7	1.00	Ref.	218	33.6	1.00	Ref.	112	13.1	1.00	Ref.
	Male	870	41.2	208	48.7	1.19	0.84, 1.70	183	36.3	1.37	0.96, 1.94	114	18.0	1.66	1.13, 2.44*
Ethnicity	White	608	28.6	164	53.4	1.00	Ref.	121	35.9	1.00	Ref.	56	13.1	1.00	Ref.
	South Asian	726	34.2	134	41.7	0.96	0.61, 1.51	126	32.1	1.11	0.70, 1.75	71	13.5	0.78	0.46, 1.34
	Black	499	23.5	120	48.8	1.06	0.66, 1.69	93	33.2	0.83	0.52, 1.33	73	21.0	1.65	0.99, 2.75
	Mixed/other	291	13.7	67	48.9	0.99	0.57, 1.74	69	44.0	1.46	0.83, 2.56	35	17.7	0.92	0.49, 1.73
Education	Primary	68	3.3	12	42.9	1.00	Ref.	5	14.3	1.00	Ref.	8	16.7	1.00	Ref.
	Secondary	392	19.4	72	41.6	0.50	0.15, 1.64	62	27.7	1.76	0.49, 6.27	28	10.6	0.36	0.12, 1.10
	College	770	37.9	208	51.9	0.75	0.24, 2.39	184	41.4	4.12	1.18, 14.36*	100	17.5	0.67	0.24, 1.91
	University	751	36.9	158	46.8	0.62	0.19, 1.99	137	35.5	4.37	1.24, 15.42*	83	15.6	0.76	0.27, 2.16
	Other	50	2.5	8	33.3	0.21	0.03, 1.33	7	25.0	1.22	0.18, 8.09	7	19.4	0.47	0.09, 2.60
Chronic disease	No	1488	72.0	343	47.9	1.00	Ref.	272	33.9	1.00	Ref.	146	13.9	1.00	Ref.
	Yes	580	28.1	124	47.5	1.04	0.69, 1.57	121	37.2	1.74	1.17, 2.59**	72	17.8	1.29	0.84, 2.00
Smoking status	Non-smoker	1515	70.6	324	47.9	1.00	Ref.	281	34.9	1.00	Ref.	148	13.9	1.00	Ref.
	Ex-smoker	296	13.8	56	40.3	0.58	0.34, 1.00*	45	30.4	0.60	0.35, 1.03	28	14.1	0.80	0.43, 1.51
	Smoker	336	15.7	97	51.9	1.23	0.79, 1.91	72	35.6	0.88	0.56, 1.38	48	19.0	1.20	0.74, 1.93
Visited dentist in last two years	No	559	24.7	93	28.2	1.00	Ref.	85	23.7	1.00	Ref.	61	13.8	1.00	Ref.
	Yes	1707	75.3	416	56.8	3.56	2.39, 5.29	344	40.0	2.57	1.72, 3.85**	175	15.5	1.34	0.85, 2.09

^a^ *N* = 746, ^b^ *N* = 882. ^c^ *N* = 1188; *p < 0.05, **p < 0.01

Observations with missing data not removed prior to analysis. Model adjusted for patients’ waiting time, whether patients could hear the Life Channel, and model accounts for clustering around GP Practices

### Knowledge in contacting dental services

Among patients who did not know how to contact an NHS dentist prior to the survey (*n* = 1088), 48.0 % (95 % CI 45.0-51.0 %) attributed the Life Channel to educating them in how to do so. This is broken down by sample characteristics and presented in Table 1. After adjustment for predictor variables, waiting time, and whether patients could hear the Life Channel, ex-smokers were less likely to attribute the Life Channel to educating them in how to contact an NHS dentist compared to non-smokers (AOR 0.58, 95 % CI 0.34-1.00). Furthermore, patients who visited a dentist in the last 2 years were more likely to attribute the Life Channel to educating them in how to contact an NHS dentist compared to those who had not visited a dentist in the last 2 years (AOR 3.56, 95 % CI 2.39-5.29).

Among patients who did not know how to contact an emergency dentist prior to the survey (*n* = 1247), 35.1 % (95 % CI 32.4-37.8 %) attributed the Life Channel to educating them in how to do so. Again, this is broken down by sample characteristics in Table 1. After adjustment, older, more educated patients, and those with chronic diseases and those who had visited a dentist in the last 2 years were significantly more likely to attribute the Life Channel to educating them in how to contact an emergency dentist.

### Intention to access dental services

Among patients who did not have any intention to contact a dentist prior to the survey (*n* = 1605), 15.2 % (95 % CI 13.4-17.0) attributed the Life Channel to intending to contact a dentist. Males were more likely than females to attribute the Life Channel to contact a dentist compared with females (AOR 1.66, 95 % CI 1.13-2.44).

### Other aspects of the life channel evaluation

The Life Channel was noticed by 85.5 % of patients prior to the survey taking place, and was the most popular waiting room activity (46.5 %). It was seen by 94.2 % of patients but heard by only 53.7 %. Among smokers, 27.6 % found out that there was a free stop smoking service in Brent, 31.5 % knew how to contact this service, and 11.6 % reported an intention to contact it. Other measures of evaluation are presented in Table 2. The Life Channel displayed good acceptability (74.7 %) and visible reach (94.2 %) to patients, although a significant proportion could not hear it well (46.3 %) and wanted to see health promotion information in other languages (43.0 %).

**Table 2:** Other measures of evaluation of the life channel

Measure	Agree or strongly agree^a^	
	%	*N*
I have learned something new about improving my health or wellbeing from seeing the Life Channel	54.5	1,194
I have learned something new about improving someone else’s health or wellbeing from seeing the Life Channel	52.4	1,126
I intend to contact another service because of the Life Channel	31.6	671
I will tell someone else about what I have seen on the Life Channel	50.7	1,050
I think that seeing the Life Channel will improve my own health and wellbeing	57.7	1,156
I would like to see the Life Channel being broadcast in a language other than English	43.0	908
While I’m waiting for my appointment I prefer reading magazines, leaflets and posters to watching TV	48.8	1,005
I like having the Life Channel on in the GP surgery waiting area	74.7	1,524

^a^Asked on a five-point Likert scale: strongly disagree, disagree, neither agree or disagree, agree, and strongly agree

## Discussion

This survey of patients attending General Practices in a multicultural area of the United Kingdom showed that among those who previously lacked knowledge, about half learnt how to contact an NHS dentist and a third learnt how to contact an emergency dentist, self-attributed to a new television-based health promotion service. Furthermore, about 15 % of patients previously not intending to contact a dentist, attributed the service to creating such an intention. This health promotion service can therefore be seen to empower patients to make more informed choices related to healthcare. We found age, gender and socioeconomic disparities regarding these outcomes, which should be borne in mind when designing future health promotion initiatives.

To our knowledge this is among the first of studies to evaluate the use of television-based dental health promotion in a General Practice waiting room, which shows promising results. We had a large, representative sample size and diverse population to identify socioeconomic differences in the knowledge and intention to access dental services. It is likely our findings are generalisable to other areas of UK that have a similarly multi-ethnic population demographic.

This study has several limitations of note. Firstly, we took a convenience sample of patients without a systematic approach that accounted for the time of day of each General Practice visit. However, this was compensated for what we believed to be a representative sample of the local area. We were unable to calculate response bias, where responders could have been more likely to engage with the survey because of noticing the Life Channel. Our survey, although pre-piloted, was not validated and thus our results should be interpreted with caution. We were not able to validate this survey due to resource limitations. It did not include questions on baseline health status with regards to oral health, obesity, diet, alcohol consumption, sexual health or drug use, nor whether the advertisements caused anxiety. In particular, the lack of data on oral health precludes us from understanding the context in which three quarters of the sample had visited a dentist in the previous 2 years, and the relative merit of including dental health as one of six key public health messages. Anxiety in dental waiting rooms are perhaps more common than in General Practice waiting rooms, and several studies have sought to alleviate anxiety in dental waiting rooms [20–22].

One barrier to implementation includes gaining co-operation from reception staff to keep the volume loud enough for patients to hear the advertisements. Indeed, anecdotal feedback from facilitators suggested that some receptionist staff turned down the volume of the Life Channel as it was noisy and disliked. This can be partially overcome by ensuring advertisements contain sufficient written text for those unable to listen to them, and by designing advertisements with input from both patients and receptionist staff. Finally, we do not have any outcome data beyond knowledge of and intention to access dental services, or whether consultation with an intermediary healthcare professional is needed to promote behaviour change. This limits our ability to recommend the Life Channel as an effective health promotion intervention. In this study we have assumed that intention to seek healthcare is a strong predictor of behaviour, however other psychosocial theories describe a multitude of predictors of behaviour change such as those described by the Health Belief Model (self-efficacy, cues to action, perceived susceptibility/severity/benefits/barriers to change) and the Stages of Change model (pre-contemplation, contemplation, preparation, action, maintenance).

## Conclusions

This study showed that television-based health promotion in a General Practice waiting room may be an effective medium to improve patients’ knowledge and intentions to access dental health services. Further research should assess the effectiveness of different health message content, including those in different languages, and explore how health promotion can be maximised to appeal to diverse population groups. Meanwhile, future research should incorporate medium to long term health outcomes, and actual access to dental services, in evaluative procedures.

## Abbreviations

AOR, adjusted odds ratio; CI, confidence interval; NHS, National Health Service; UK, United Kingdom
